# Psychological and Emotional Effects of Digital Technology on Children in COVID-19 Pandemic

**DOI:** 10.3390/brainsci11091126

**Published:** 2021-08-25

**Authors:** Pierpaolo Limone, Giusi Antonia Toto

**Affiliations:** Department of Humanistic Studies, University of Foggia, 71121 Foggia, Italy; pierpaolo.limone@unifg.it

**Keywords:** digital technology, brain condition, neuropsychological effects, COVID-19

## Abstract

COVID-19 has caused obstacles in continuing normal life almost everywhere in the world by causing the implementation of social distancing and eventually imposing the lockdown. This has become the reason for the increase in technology usage in daily life for professional work as well as for entertainment purposes. There has been an increased prevalence of technology usage in adolescents and children during lockdown leaving its impact on their lives either in a positive or negative aspect. The overall documented percentage increase of technology usage in children was about 15%, of which smartphone usage has 61.7% of prevalence. Disturbance in brain functioning is suggested to be originated by compromise of neuroplasticity of the nerves. The radiofrequency (RF) radiations emitting from the smartphone are of doubtful concern as a brain tumor risk factor in children. The increased usage can have effects on brain functioning that will compromise sleep and cognitive abilities and develop risk for certain mental illnesses including, but not limited to, depression, anxiety, Alzheimer’s disease, and attention-deficit/hyperactive disorder (ADHD). Despite being a threat for developing mental illness, video games are proven to reduce depression and anxiety, and increase creativity, skills, and cognition in children. The increased usage of technology can have a positive and negative impact on the mental development of adolescents and children depending on the trends in the usage. However, parents should be monitoring their children’s mental health and behavior in these difficult times of pandemic.

## 1. Introduction

The outbreak of novel coronavirus, SARS-CoV-2, has posed a greater concern to the world due to it being new to the healthcare community and the lack of any specific medical treatment available on the spot for COVID-19 patients. SARS-CoV-2 initially affects the lungs and respiratory system before spreading its infection and inflammatory responses to the other parts of the body, i.e., kidney, heart, and liver problems [1]. The virus enters lung cells through angiotensin-converting enzyme-2 (ACE2) and starts replicating there before producing symptoms in a span of 7 to 14 days [2]. The most common symptoms of COVID-19 include fatigue, fever, dry cough, headache, dyspnea [3]. The mortality rate of the COVID-19 patients in the world is reported to be 17.1% as of March [4]. The mortality rate in non-critical patients was found to be 11.5% while the mortality rate in critically ill COVID-19 patients is 40.5% [4]. Due to the alarming levels of spread and severity all over the world, WHO declared the COVID-19 as a pandemic [5]. Following the concern of increased cases and severity along with its declaration as a pandemic, officials of governments all over the world took drastic measures along with imposing lockdown during the pandemic to reduce the spread of the virus [6] Although lockdown proved to be effective to reduce the spreading of virus, there’s been another rising concern in the form of behavioral, emotional, psychological, and neurological effect of lockdown as well as worsening of the pre-existing neurological disorders in this pandemic [7,8]. Upon imposing the lockdown, there had been reports of the occurrence of anxiety, somatic problems, obsessive compulsive, post-traumatic stress, and thought problems in children ranging from 1.5 to 18 years of age [7]. It has been documented that children are fearful of the pandemic and feel anxious due to the quarantine and also feel isolated because of prolonged closure of parks, schools, theaters, and lack of playing outdoor games [9,10]. Previous studies have observed anxiety, depression, irritability, inattention, mood swings, and poor sleep quality as a common problem during the quarantine in the COVID-19 pandemic [9,11]. Students have also been observed to suffer due to interrupted education, and an uncertain future [12]. The use of smartphones and other technologies during the pandemic has been increased noticeably due to quarantine and nowhere to go. Not only parents, but children also, increased their technology use, i.e., in the purpose of gaming, online classes, time passing including social media use [13]. Along with other factors to cause psychological, and neurological effects on children during a pandemic, the overuse of technology is also considered a concerning factor to have these effects on the mental health of children [13]. According to one study, there had been 15% increase in technology use in participants who were using it ‘all the time’. This increase in technology use is documented as the risk factor of developing psychological conditions [14]. In the summer of 2021 (June), different trends began to spread (loosening of restrictions, quota schedules, end of lockdown) all over the world united by two macro characteristics: the spread of the delta variant [15] and the younger age of coronavirus cases [16]. The analysis of the latter phenomenon is being studied by medicine since the causes of this further spread are multifactorial (less vaccinated age group, end of restrictions, etc.). Naturally, the spread of such news through the media and the circumscription of some restrictions in some national realities are reproposing the problem of a widespread anxiety-inducing climate. The conscious and controlled use of technology in school contexts can be a valid proxy for the positive use of technologies in the lives of children and adolescents [17].

### Aim and Objective

The rate of technology use in the COVID-19 pandemic has been increased drastically to extent of its overuse in daily life. As much as technology is being used in a positive perspective during a pandemic, it is also being convicted of negative impact on neurological and psychological functions. The purpose of this study is to review systematically the effects of technology on the neurological functions of children in the COVID-19 pandemic.

## 2. Methodology

### 2.1. Search Strategy

Different databases were used to search the studies for the current review, these databases included “Google Scholar”, “PubMed”, “Cochrane Library”, “ScienceDirect”, and “ResearchGate”. The keywords for searching included “smartphone”, “COVID-19”, “Children”, “Pandemic”, “Technology use in COVID-19”, “Effect of technology and children in a pandemic”, “Psychological effects of COVID”, “Emotional effects of the pandemic in children”, and “Neurological effects of lockdown”. The studies were double-checked and filtered by two of the authors. The relevant studies to the current review were included while the irrelevant ones were discarded.

The articles were selected on the basis of three guiding ideas: “effect of digital technology on children in a pandemic”, “psychological and emotional effects of COVID”, and “neurological effects of lockdown”. Regarding the inclusion and exclusion criteria, the articles were selected in peer-reviewed English journals that aimed to describe or evaluate the dimensions and variables expressed vis-à-vis the research topic mentioned above (screening). Publications that did not deal with the topic in relation to the age group were excluded, as were those publications whose full text (relevance) was not found. Book chapters, books, news articles, and legal reports were also excluded. A qualitative synthesis of the most relevant information was also conducted with comparisons between the various publications; this was done without carrying out a quantitative analysis in the meta-analysis format.

**Figure 1:**
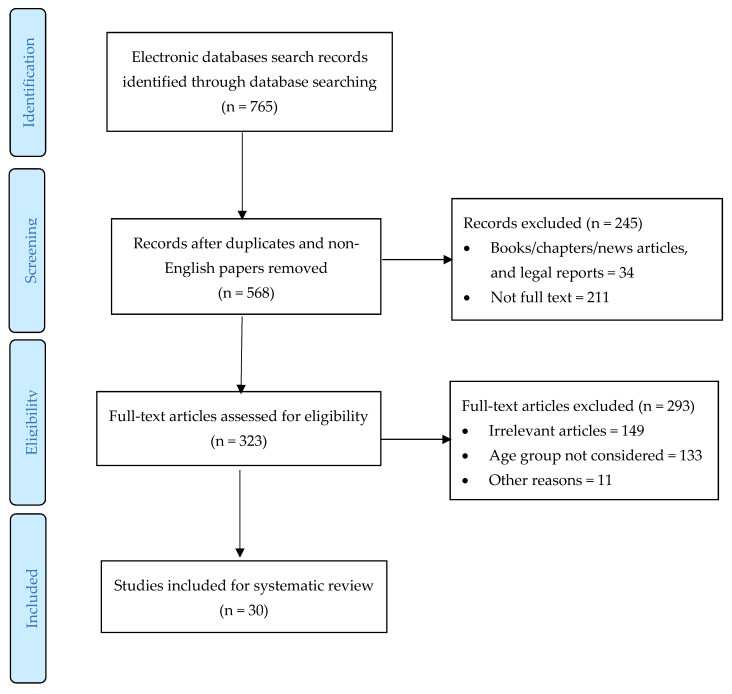
PRISMA flow chart of the selection process.

The process of including studies in the systematic review is described in Figure 1. After the elimination of duplicates and articles in languages other than English, the search identified 568 studies consistent with the research parameters. After excluding the publications that were not relevant (n = 245) and those that had to be excluded because their content or age led them to not coincide with the research sample (n = 160), 30 were left that met the inclusion criteria.

### 2.2. Population Selection

The population for the current review is children and adolescents to measure the effect of technology. Studies including children with the age of lesser than 18 years are included in the study following the objectives of the current study (Table 1). To extract the data from the articles, the following coding process was followed: (1) author/authors and year of publication, (2) title of the research, (3) place/country of publication, and (4) key ideas of the research. In order to establish the methodological quality of this study, the reliability was determined based on the survey and selection of the Kohen’ Kappa statistical index (for agreement) for two evaluators, according to which 0.81–1.00 represents an almost perfect agreement [18]. For the extraction and selection of the data a value of K = 0.82 was obtained.

## 3. Prevalence of Technology Use in Children

Most of the references in this article (46.66%) are from the year 2020–2021, a sign of the interest in the topic of the effects of digital technology on cognition and the dedication of constant international academic research over the last 10 years that led up to a peak in research activity in the three years before the pandemic. From the analysis of the results (Table 2), it was possible to identify four main research lines: prevalence of technology use in children, neurological changes in children, brain conditions and diseases associated with smartphone use in children, and impact of television and videogames.

Prevalence of technology usage including smartphone has been increased noticeably since the pandemic and lack of outdoor activities due to home confinement of the children in lockdown. The overall increase in prevalence of technology use during COVID-19 pandemic is reported to be 15% [14]. One study has found the 16.4% of prevalence of smartphone use in children during the pandemic and is labeled as “problematic prevalence” [47]. The study, conducted in 217 participants, reported the mean duration of digital device usage was 3.9 h ± 1.9 h which was more than the pre-COVID era (1.9 h ± 1.1 h with *p*-value < 0.0001). 36.9% of participants were using digital devices more than 5 h a day as compared to pre-COVID era which was 1.8% of participants. Among the digital devices, the most common device to be used was smartphone with the prevalence of 61.7% that is worth of noticeable [19]. This rising prevalence is the indicator of developing the internet addiction, gaming disorder, anxiety, depression, irritability sleep disturbance, and poor health [19,47]. Education was heavily impacted by the COVID-19 pandemic. Children are experiencing restlessness and stress in adapting to distance learning. YouTubeKinds is a tool that has enabled the potential to increase informal learning and entertainment opportunities for children [20]. As for adolescents among the factors that influence subjective well-being (studied in an American context) during the first months (April–August 2020) of the coronavirus pandemic. Experimental studies have focused [21] on a possible higher incidence of addiction to technologies and the network in adolescents because they spend more time with the virtual. The study by Pitt and Hock [21] showed that the total time teens spend with technology has less impact on daily fluctuations in well-being than the satisfaction and meaning that comes with their use of technology. During 2020 teenagers due to the change in daily lifestyle experienced feelings of fear, discouragement and anxiety which strongly affected their mood. In the study by [22] interviewed adolescents (aged 12 to 18) reported using this period to acquire new skills and to practice physical activity at home. The use of technology was predominant for both recreational and educational purposes. Despite the strong psychological impact of quarantine, adolescents showed good levels of resilience. Technology played a crucial role during the quarantine, in fact, the daily use of technological devices has increased.

## 4. Neurological Changes in Children

Neuroplasticity refers to the structural and functional changes in the brain caused by neuron development with passage of time leading to the experience-dependent change [23]. The connection between neurons increases more rapidly in childhood than adulthood, that is why early experiences have huge impact on brain development [24]. The use of technology affects the neuroplasticity in children and adolescent resulting in change that might be transient or permanent [25]. Early infancy and adolescence are crucial years for brain growth and reorganization; thus, experiences and environmental variables can have a significant impact on future brain functioning [26,27].

## 5. Brain Conditions and Diseases Associated with Smartphone in Children

The addiction of technology has risen up to noticeable point and it keeps rising in the children and adolescents of different communities around the globe. Among the technologies, most widely used machine is smartphone. There are many worrisome conditions that are caused by the smartphones as mentioned in the aforesaid section. Smartphones are said to emit radiofrequency (RF) capable of reaching the brain leading to the unwanted events. The frequency range of 30 KHz–300 GHz is suggested to be the possible human carcinogen [28]. This RF emission is documented for developing the brain tumor risk in children and adolescents. It was found that the brain region exposed to RF radiation are prone to develop the glioma and acoustic neuroma for tumors in children and adolescents [29]. The risk is reported to be highest in population of <20 years [30]. Children and adolescents are more exposed to RF radiations of wireless phone due to smaller heads, higher conductivity, and thinner skulls than the adults [31]. These factors contribute in the higher absorption of RF radiation through children’s brains [31]. Smartphone also cause the sleep disturbance due to the RF radiations [32]. The use of cellphones was associated with the lower concentration of Beta-trace protein (lipocalin-type prostaglandin D synthase) which synthesizes the essential sleep-promoting neurohormone named as prostaglandin D [33]. Along with the brain tumor risk, WHO reported the wireless phone being the health risk including, attention deficiency, impaired cognition, impaired learning, sleep disruptions, and sensitivity to stress. Other noteworthy conditions are Alzheimer’s disease, “got dementia”, depression, anxiety, and risk for developing any possible neurodegenerative condition [32]. There is positive and negative evidence regarding the use of technology in relation to neurodevelopment or neuroplasticity: among the negative effects, excessive exposure to the screen can influence brain development in negative ways [34], it can increase the risk of cognitive, behavioral, and emotional disturbances in adolescents and young adults (or the risk of dementia in old age).

VRT (virtual reality technology)-based rehabilitation is increasingly used to encourage patient recovery in the physical and cognitive domains [35]. The advantages of using VRT in neurorehabilitation are the possibility of recovering a compromised function as a way to stimulate [36] neuronal reorganization and the induction of neuroplasticity (to maximize motor learning and neuroplasticity) and regain functions and abilities (even partially) by interacting with a virtual reality environment (VRE). The reference literature suggests that the alleged danger of the use of smartphones [48,49], in particular as a risk factor for the development of brain tumors, is in fact still debated and, even if the fear can be justified, we have no evidence that the danger is real [50,51,52].

## 6. Impact of Television and Videogames

Television has been around for a long time. There are several studies that have been done but their sample sizes are rather small to reach any conclusions for a large population. However, television has a huge impact on children from the day 1 because everyone has it in his/her home. Studies have associated the television with the attention problem in adolescence and children [37]. It is also documented that watching television before 3 years of age may have noticeable effect on cognitive functioning of the child [38]. This pandemic has been the reason for increased use of television among children. Analyses of how children’s brains react to television use are scarcer than those concerning cognitive or behavioral outcomes, and causality remains difficult to ascertain [24].

Gaming has become an essential part of the life of children, sometimes leading to the “Internet Gaming disorder” or “Gaming disorder” due to their addictive propensity. Gaming is suggested to have great impact on human reward system (through dopaminergic pathways), impulse control, and sensorimotor co-ordination [39]. Game playtime and frequency of play has increased rapidly during the events of COVID-19 [40]. Playing videogames in the pandemic embarked some positive results in reducing depression, anxiety, improving mental health, and combating loneliness [41]. Videogames are also documented to stimulates or improve the cognitive function as concluded by the recent research [41]. According to the previous study on videogames in COVID-19, videogames have more positive impacts as aforementioned than the negative impact, and the included negative impacts were relatively least impactful on daily life as they were only complaining about the wastage of time [41]. The use of smartphones and other technologies during the pandemic has been increased noticeably due to quarantine. One study found the daily smartphone and tablet exposure of 2 out of 3 children under 48 months in Spain [42]. The children increased their technology usage during COVID-19 pandemic, i.e., in the purpose of gaming, online classes, and passing time including social media use [13]. As already mentioned, the advent of the pandemic has influenced the behavioral pattern of the younger generation in relation to health, lifestyle, and physical activity level to screen addiction, causes various diseases, social problems, poor school performance and negatively affects on indicators of their physical and mental health [43]. In fact, there was an increase in the time spent in front of screens or a hyper-connection to the internet. In general, the associated sedentary lifestyle. In a study conducted in China [44], the prevalence of PSU (problematic smartphone use) was 43.3% in the overall sample, with 41.9% in women and 45.5% in men. To date, little qualitative research has been conducted with adolescent smartphone users when this is particularly problematic or excessive use. The results of the study by Conlin and Sillence [45] demonstrated the complexity of discriminating between functional and fun smartphone use from problematic use in an era where smartphones are so deeply present in modern life. Among the problematic aspects reported was the need to have their phones in the immediate vicinity even at night, the anxiety of having lost their phones or the distraction from their phones while getting to know other people. The sense of comfort and evasion provided by smartphones seems to help avoid unpleasant thoughts, emotions or experiences by providing a variety of new stimuli. When the degree of internet addiction becomes high, subjects (usually young adults) show a low level of inhibitory (psychophysiological) control, while subjects who have a lower degree of dependence on both a computer and the Internet have a more flexible nervous system, which is indicated by the highest level of inhibitory control [46]. From this study, it can be assumed that less Internet dependent students will be able to adapt to a rapidly changing environment.

## 7. Conclusions and Future Recommendations

Increased technological usage during the pandemic has its positive and negative impacts, depending on the usage. As much as smartphones are way of escaping loneliness in lockdown, they are also responsible for causing serious mental illness including depression, anxiety, sleep irritability, and cognitive impairment. The RF radiations emitting from the smartphone are of doubtful concern as brain tumor risk factor in children. Further on, although television usage might be not as much as the smartphone, it also has its effects on children up to some extent. Videogames are proven to be stress relief tools for the children as well as adults. Videogames are claimed to reduce depression and anxiety, and increase creativity, cognition, and skills [53].

Parents should be checking on their children for any possible negative impact of increased usage of technology. Individuals who are sensitive to stress or prone to develop depression, anxiety should be encouraged to make their distance from the daily news because of negativity [54]. Parents are suggested to introduce productive and creative games in their homes and should motivate their children.

## Figures and Tables

**Table 1:** Search strategy.

Database	Keywords	Population
Google Scholar, PubMed, Cochrane Library, ScienceDirect, ResearchGate.	Smartphone, COVID-19, Children, Pandemic, Technology use in COVID-19, Effect of technology and children in a pandemic, Psychological effects of COVID, Emotional effects of the pandemic in children, Neurological effects of lockdown.	Children and adolescents: studies including children with the age of lesser than 18 years are.

**Table 2:** Results.

Authors (Year)	Title	Nation(s)	Core Concept
Ammar, et al., (2021) [14].	Effects of home confinement on mental health and lifestyle behaviors during the COVID-19 outbreak: insights from the ECLB-COVID19 multicenter study.	Western Asia, North Africa, Europe	Prevalence of technology use in children.
Mohan, et al., (2021) [19].	Prevalence and risk factor assessment of digital eye strain among children using online e-learning during the COVID-19 pandemic: Digital eye strain among kids (DESK study-1).	India	
Temban, et al., (2021) [20].	Exploring informal learning opportunities via youtube kids among children during COVID-19.	India	
Pitt, & Hock, (2021) [21].	The kids are/not/sort of all right* technology’s complex role in teen wellbeing during COVID-19.	USA	
Salzano, G et al., (2021) [22].	Quarantine due to the COVID-19 pandemic from the perspective of adolescents: The crucial role of technology.	Italy	
Mundkur, (2005) [23].	Neuroplasticity in children.	India	Neurological changes in children
Gottschalk, (2019) [24].	Impacts of technology use on children: Exploring literature on the brain, cognition and well-being, 2019.	World	
Bavelier, et al., (2010) [25].	Children, wired: For better and for worse	USA	
Irwin, L. (2007) [26].	Early child development: A powerful equalizer. Final report for the World Health Organization’s Commission on the social determinants of health.	World	
Petanjek, et al., (2011) [27].	Extraordinary neoteny of synaptic spines in the human prefrontal cortex.	France	
IARC 1988 [28].	International Agency for Research on Cancer. IARC monographs on the evaluation of carcinogenic risks to humans. v. 42: Alcoholic drinking).	France	Brain Conditions and diseases associated with smartphone in children
Hardell, & Carlberg, (2015) [29].	Mobile phone and cordless phone use and the risk for glioma–Analysis of pooled case-control studies in Sweden, 1997–2003 and 2007–2009.	Swezia	
Carlberg, & Hardell, (2014) [30].	Decreased survival of glioma patients with astrocytoma grade IV (glioblastoma multiforme) associated with long-term use of mobile and cordless phones.	Swezia	
Gandhi, et al., (2012) [31].	Exposure limits: The underestimation of absorbed cell phone radiation, especially in children.	USA	
Hardell, (2018) [32].	Effects of mobile phones on children’s and adolescents’ health: A commentary.	German	
Söderqvist, et al., (2012) [33].	Use of wireless phones and serum β-trace protein in randomly recruited persons aged 18–65 years: A cross-sectional study.	Sweden	
Neophytou, et al., (2019) [34].	Effects of excessive screen time on neurodevelopment, learning, memory, mental health, and neurodegeneration: A scoping review.	North America, (including Canada, and the USA, Europe, Asia, Australia, New Zealand, and the Middle East)	
Naro, & Calabrò, (2021) [35].	What do we know about the use of virtual reality in the rehabilitation field? A brief overview.	Italy	
Nizamis, et al., (2021) [36].	Converging robotic technologies in targeted neural rehabilitation: A review of emerging solutions and challenges.	Greece	
Landhuis, et al., (2007) [37].	Does childhood television viewing lead to attention problems in adolescence? Results from a prospective longitudinal study.	New Zealand	Impact of television and videogames
Zimmerman, & Christakis, (2005) [38].	Children’s television viewing and cognitive outcomes: a longitudinal analysis of national data.	USA	
Gottschalk, (2019) [24].	Impacts of technology use on children: Exploring literature on the brain, cognition and well-being,	World	
Weinstein, & Lejoyeux, (2015) [39].	New developments on the neurobiological and pharmaco-genetic mechanisms underlying internet and videogame addiction.	World	
Marston, & Kowert, (2020) [40].	What role can videogames play in the COVID-19 pandemic?	UK	
Barr, & Copeland-Stewart, (2021) [41].	Playing Video Games During the COVID-19 Pandemic and Effects on Players’ Well-Being	World	
Cartanyà-Hueso, et al., (2021) [42].	Smartphone and tablet usage during COVID-19 pandemic confinement in children under 48 months in Barcelona (Spain).	Spain	
Agafonov, et al., (2021) [43].	Actual problems of physical development of children in the age of digital technologies.	Russia	
Zhang, et al., (2021) [44].	Problematic smartphone use during the COVID-19 pandemic: Its association with pandemic-related and generalized beliefs.	Cina	
Conlin, & Sillence, (2021) [45].	Exploring british adolescents’ views and experiences of problematic smartphone use and smartphone etiquette.	UK	
Merenkova, et al., (2021) [46].	Psychophysiological markers of students’ internet addiction in the era of digitalization	Russia	
